# Sleep and physical activity in patients with newly diagnosed bipolar disorder in remission, their first-degree unaffected relatives and healthy controls

**DOI:** 10.1186/s40345-020-00181-6

**Published:** 2020-06-01

**Authors:** Nikolaj Folke la Cour Karottki, Klara Coello, Sharleny Stanislaus, Sigurd Melbye, Hanne Lie Kjærstad, Kimie Stefanie Ormstrup Sletved, Lars Vedel Kessing, Maj Vinberg

**Affiliations:** 1 Copenhagen Affective Disorders Research Centre (CADIC), Psychiatric Center Copenhagen, Department O, 6243, Rigshospitalet, Blegdamsvej 9, 2100 Copenhagen, Denmark; 2 Faculty of Health and Medical Sciences, University of Copenhagen, Copenhagen, Denmark; 3 Psychiatric Research Unit, Psychiatric Centre North Zealand, Hillerød, Denmark

**Keywords:** Bipolar disorder, Sleep, Physical activity, Unaffected relatives

## Abstract

**Background:**

Sleep disturbances are a central feature in bipolar disorder (BD) that often persist in remission and seem to be present also in unaffected first-degree relatives (UR) of patients with BD, presenting a possible risk factor for later onset of BD. However, it is unknown if these disturbances are associated with unhealthy life-style as reflected in low levels of physical activity. We investigated sleep disturbances and physical activity levels in patients with newly diagnosed BD in full or partial remission, their UR and healthy controls (HC).

**Methods:**

Sleep patterns and physical activity were compared in 227 patients with newly diagnosed BD, 76 UR and 148 HC. The Pittsburgh Sleep Quality Index (PSQI) and the International Physical Activity Questionnaire (IPAQ) were used to assess sleep disturbances and physical activity, respectively.

**Results:**

In sex- and age-adjusted analyses, patients with BD exhibited more sleep disturbances and lower physical activity compared with UR and HC, respectively. Unaffected relatives reported significantly longer sleep latency and a non-significant trend towards more overall sleep disturbances compared with HC.

**Conclusions:**

Sleep disturbances and less physical activity are present in patients with newly diagnosed BD in partial or full remission. Individuals at familiar risk of BD reported longer sleep latency and similar physical activity compared with HC. Further prospective studies are needed to clarify whether these discrete sleep disturbances act as risk factor for later onset of BD and whether increased physical activity in high-risk individuals may act as a protective factor against development of psychiatric illness.

## Background

Bipolar disorder (BD) is a recurrent mood disorder with episodes of depression and mania. It is often associated with functional disability, decreased quality of life and reduced life expectancy (Rosa et al. 2014; Kessing and Andersen 2017). The mean age of onset is mid to late twenties in Europe and the estimated lifetime prevalence is thought to be 1–2% (Pedersen et al. 2014; Post et al. 2008). A substantial hereditary component is seen in families of patients with BD with the approximate lifetime risk of BD in first-degree relatives being 5–10% and monozygotic co-twins 40–70% (Craddock and Sklar 2013; McGuffin et al. 2003). Studies show that up to 50% of first-degree relatives of patients with BD will develop a mood disorder or another psychiatric illness (Mesman et al. 2013; Duffy et al. 2015). However, knowledge of the specific factors that run cross generations is needed to tailor a more integrated preventive approach. Further, there is a latency of approximately 6 years from onset to initial treatment of BD (Dagani et al. 2017). Yet, early treatment seems to hinder a more severe progression of the disorder (Bender and Alloy 2011; Shapero et al. 2017; Kessing et al. 2014) and it is thus crucial to discover robust risk factors and valid biomarkers.

Central features in BD are circadian rhythm abnormalities including sleep disturbances (Gold and Sylvia 2016; Takaesu 2018) characterized by difficulty falling asleep, maintaining sleep and early morning awakening leading to dissatisfaction with sleep quantity and quality (Buysse et al. 2017). These changes in sleep pattern often persist after manic and depressive episodes and they further seem to be present in unaffected first-degree relatives (UR) of patients with BD as well, presenting a possible risk factor of developing BD (Keskin et al. 2018; Melo et al. 2016a; Ritter et al. 2015). Unaffected first-degree relatives of patients with BD might have a latent-stage or risk stage BD (Kapczinski et al. 2009, 2014; Berk et al. 2017) due to a possible shared biological underpinning. A systematic review including 30 studies and 5778 high-risk individuals (Melo et al. 2016a) showed more sleep disturbances and poor sleep quality in high-risk individuals cross-sectionally. Five prospective studies were also included (n = 3759), four of which investigated offspring of parents with BD (follow-up periods ranged from 4 to 16 years) and these revealed that poor sleep quality, trouble falling asleep, early morning awakening, night-time awakenings and inadequate sleep could be an indicator of later onset of BD (Melo et al. 2016a). This is in line with a recent review focusing on sleep alterations anticipating the onset of BD including 17 studies both prospective (n = 6) and retrospective (n = 11), concluding that sleep alterations also seem to appear long before the onset of BD. Nevertheless, sleep disturbances have not been investigated in newly diagnosed BD and their healthy first-degree relatives (Pancheri et al. 2019).

Physical activity is widely known to reduce risk of all-cause mortality, cardiovascular disease and cancer-related mortality compared to sedentary behaviour (Andersen et al. 2000; Kodama et al. 2009; Lear et al. 2017; Piercy et al. 2018) and sleep disturbances are associated with a risk of all-cause mortality and cardiovascular events (Yin et al. 2017). Bipolar disorder is associated with comorbid somatic disorders mainly cardiovascular disease, obesity and non-insulin dependent diabetes (Evans et al. 2005). Putative reasons for these conditions and the increased mortality observed in patients with BD (Correll et al. 2017) include a more sedentary lifestyle (Vancampfort et al. 2016; Kilbourne et al. 2007) as well as abnormal sleep patterns (Yin et al. 2017; Cappuccio et al. 2010). The increased cardiovascular risk seems further to be present in youth with BD (Goldstein et al. 2015) and in young adults with newly diagnosed bipolar disorder (Coello et al. 2019). Consequently, it is highly interesting to investigate physical activity and sleep patterns in BD and preceding BD by studying patients with newly diagnosed BD and their first-degree relatives.

Overall patients with BD and UR seem to express a disturbed sleep pattern, however, whether this is associated with an unhealthy life-style reflected in decreased physical activity has not previously been studied in patients newly diagnosed with BD and their UR.

This study aimed to investigate sleep disturbances (and patterns) and patterns of physical activity among patients with newly diagnosed BD in full or partial remission, UR and HC. We hypothesized that patients with BD in full or partial remission would report more sleep disturbances and less physical activity than HC and that the UR would show an intermediary pattern in both outcomes.

## Materials and methods

### Study design

The Copenhagen Affective Disorder Clinic is an outpatient clinic that covers the Copenhagen Capital region catchment area of 1.6 million inhabitants and offers service for patients with newly diagnosed BD. Patients referred to the Copenhagen Affective Disorder Clinic as newly diagnosed with BD or having a first episode of (hypo)mania were routinely invited to participate in the Bipolar Onset Study (BIO) (Kessing et al. 2017). In the present cross-sectional study, we compared self-reported sleep patterns and physical activity levels across the three groups (patients with BD, UR and HC). Participants were recruited from June 2015 to December 2018.

#### Patients with bipolar disorder

All patients with newly diagnosed BD included in the BIO study were recruited from the Copenhagen Affective Disorder Clinic, an outpatient clinic covering the entire catchment area of the Capital Region of Denmark. Inclusion criteria were newly diagnosis with an ICD-10 diagnosis of BD or single (hypo)manic episode and age 18–70.

#### Unaffected relatives

First-degree relatives, siblings or children, to the patients included in the BIO-study, were recruited after permission from patients with BD, aged 15–50 years, with no history of substance abuse, psychotic illness or mood disorders according to the ICD-10 diagnostic system (International Statistical Classification of Diseases and Related Health Problems 10th Revision (ICD-10)-WHO 2016) were invited to participate upon obtaining consent from the patients.

#### Healthy controls

Healthy control persons matched on sex and age to the participants with BD were recruited from the Blood Bank at Rigshospitalet, Copenhagen, Denmark. Exclusion criteria included a personal or first-degree relative with a history of psychiatric disorders requiring treatment.

#### Psychiatric assessment

Diagnoses were confirmed using the Schedules for Clinical Assessment in Neuropsychiatry (Wing et al. 1990) and clinical assessments using the Hamilton Depression Scale-17 items (HAMD-17) (Hamilton 1960) and the Young Mania Rating Scale (YMRS) (Young et al. 1978). Only patients in remission or partial remission defined as a score ≤ 14 at the HAMD-17 and ≤ 14 at the YMRS were included in the present study. All participants completed three self-reported questionnaires: the Pittsburgh Sleep Quality Index (PSQI), the International Physical Activity Questionnaire (IPAQ) and the Copenhagen City Heart Study (CCHS) physical activity questionnaire at the day of the assessment.

#### Physical assessment

Lightly dressed and without shoes, height and weight was measured using a stadiometer and a calibrated floor scale.

### Questionnaires

The PSQI (Buysse et al. 1989) covers the last month and includes items such as duration of sleep, sleep latency, sleep disturbances, daytime dysfunction due to sleepiness, sleep efficiency, sleep medication and perceived quality of sleep. A higher score shows worse self-evaluated sleep, and a score of ≥ 5 implies poor sleep quality.

The IPAQ short form (Craig et al. 2003) reflects physical activity the preceding 7 days and is a questionnaire to address level of physical activity and sedentary behavior. The IPAQ provides information regarding time spent in four intensity levels: (1) vigorous-intensive activity, (2) moderate-intensity activity, (3) walking and (4) sedentary. Participants were asked to recall activity level for the past 7 days. Summary measures of overall self-reported physical activity are reported as a continuous variable metabolic equivalent task (MET minutes a week), representing the energy expended during the physical activity. Higher scores correspond to higher activity.

The score is an estimated value of MET-minutes, the kilocalories that a person weighing 60 kg would expend. We then calculated the corresponding MET-minutes for everyone’s weight, using the following formula:

$$Total\;METminutes*\left( {\frac{Weight}{60}} \right).$$

In accordance with the guidelines for IPAQ scoring, we truncated values above 180 min/day and excluded values exceeding 16 h/day of activity (IPAQ Group 2005).

As a secondary measurement of physical activity, we included the CCHS (Aguib and Al Suwaidi 2015) physical activity questionnaire. The CCHS is a population study of more than 24.000 men and women in Denmark, of 20 years or older with a follow-up time of 35 years. The focus is on cardiovascular disease and its risk factors including exercise. In the physical activity questionnaire used in CCHS, participants indicate one of four categories best characterizing their level of activity, ranging from less than 2 h of low activity per week to more than 4 h of strenuous activity per week.

### Statistical analyses

The descriptive data were first explored for normality and categorical data were analyzed using the Chi-squared test. Continuous data was analyzed either using Student’s T-test (parametric) or Mann–Whitney U-test (nonparametric) for two independent groups. Continuous data was described as median and quartiles if the assumptions of normality were not met. Using a general linear mixed model in SPSS version 26, we compared sleep disturbances and physical activity levels across the three groups with familial relationship as random factor, accounting for the relationship between family-related individuals, and age and sex as covariates. To check for the influence of mood symptoms, we included HAMD-17 and YMRS scores and smoking (package years) as covariates in additional analyses. To avoid overcorrection, we excluded sleep items when adjusting the PSQI. The significance threshold was set at p ≤ 0.05. Spearman’s rank-order correlation for nonparametric data post hoc analyses were done between groups (patients with BD, UR and HC) to check for correlations between sleep, physical activity, age, BMI and education levels. Within the BD group, we further examined correlations with age of onset, time of untreated BD, number of episodes and medication use. Untreated BD was defined as time from first hypomanic, manic or mixed episode to time of diagnosis. Finally, we compared the physical activity levels between the three groups using the CCHS to investigate whether it would provide the same pattern as the IPAQ.

### Ethical

The study protocol was approved by the Committee on Health Research Ethics of the Capital Region of Denmark (Protocol No. H-7-2014-007) and the Danish Data Protection Agency, Capital Region of Copenhagen (RHP-2015-023). All participants provided written informed consent. The study complied with the Declaration of Helsinki principles (Seoul, October 2008).

## Results

### Demographic and clinical characteristics

We included 451 participants, 227 patients newly diagnosed with BD in full or partial remission, 78 UR and 148 HC. The clinical and demographic variables are shown in Table 1. The age distribution was similar in the BD and HC groups (median [interquartile range]: BD = 28 [24–36] years vs. HC = 27 [24–36] years), while the UR group was younger (26 [22–31] years, p = 0.010). There were no statistically significant differences in sex distribution between the three groups. Education attainment differed statistically significantly, with HC having 16 years [15–17] vs. patients with BD 15 years [12–17] (p ≤ 0.001) of education. There were no statistically significant differences in BMI between the groups. Patients with BD had a median age of illness onset of 17 [14–22] years and the median duration of untreated BD was 3 [1–10] years.

**Table 1:** Clinical and demographic variables in patients with bipolar disorder (BD), unaffected relatives (UR) and healthy controls (HC)

	BD^1^	UR^2^	HC^3^	p-value
Number (n)	227	76	148	
Age (years)	28 [24–36]	26 [22–31]	27 [24–36]	0.01^1–2^ 0.921^1–3^ 0.28^2–3^
Sex (Female n (%))	135 (59.5%)	43 (56.6%)	89 (60.0%)	
Alcohol (units/week)	3 [0–7]	2 [1–7]	5 [2–8]	0.918^1–2^ 0.000^1–3^ 0.001^2–3^
Smoking (package years)	1.9 [0–6.8]	0 [0–0.4]	0 [0–0]	0.000^1–2^ 0.000^1–3^ 0.132^2–3^
Education (years)	15 [12–17]	15 [13–17]	16 [15–17]	0.165^1–2^ < 0.001^1–3^ 0.069^2–3^
BMI (kg/m^2^)	24.6 [22.2–27.2]	23.9 [21.5–26.4]	23.7 [22.1–26.2]	0.108^1–2^ 0.131^1–3^ 0.623^2–3^
HAM-D17	7 [3–10]	2 [0–3]	1 [0–2]	0.000^1–2^ 0.000^1–3^ 0.004^2–3^
YMRS	2 [0–5]	0 [0–2]	0 [0–2]	0.000^1–2^ 0.000^1–3^ 0.708^2–3^
Age of onset (years)	17 [14–22]	–	–	–
Total affective episodes	12 [6–23]	–	–	–
Illness duration (years)	9 [5–15]	–	–	–
Untreated bipolar disorder (years)	3 [1–10]	–	–	–
Antiepileptics	110 (52.6%)	–	–	–
Antipsychotics	93 (44.9%)	–	–	–
Antidepressants	102 (49.0%)	–	–	–
Lithium	79 (38.0%)	–	–	–
Combination treatment	2 [1–3]	–	–	–

Continuous variables shown as median [interquartile range] and categorical values as n (%). p-values denoted as the groups between which the comparison is made. Age of onset is the age at the time of the first affective episode. Illness duration is defined as the duration between first affective episode and inclusion in the study. Untreated bipolar disorder: time from first hypomanic, manic or mixed episode to time of diagnosis

*HAM-D17* Hamilton Rating Depression Scale, 17 items, *YMRS* Young Mania Rating Scale

^1^Patients with bipolar disorder (BD)

^2^Unaffected first-degree relatives (UR)

^3^Healthy control persons (HR)

As expected, HAMD-17 and YMRS scores were statistically significantly higher in the BD group compared with UR and HC (p = 0.001), and UR also had higher HAMD-17 scores (2 [0–3] vs. 1 [0–2], p = 0.004) than HC.

### Sleep

Patients with BD had a total PSQI median [interquartile range] of 8 [5–11], indicating sleep disturbances even when in remission and differed statistically significantly from the UR group (5 [3–7], p  ≤ 0.001) and the HC group (4 [3–6], p ≤ 0.001), see Table 2. At item level, all the PSQI subitems significantly differed between BD and HC, see Table 2. The differences in PSQI score remained statistically significant also when adjusting for depressive and manic symptoms and when adding smoking as a covariate (results not presented in detail). Further, removing the patients with BD with HAMD17 or YMRS above seven, leaving 99 patients with BD, the results, concerning PSQI and the subitems remained almost the same (results not presented in details) except that the significant differences between patients with BD and the UR were no longer significant concerning sleep disturbances (p = 0.185) and overall sleep quality (p = 0.078).

**Table 2:** Pittsburgh Sleep Quality Index (PSQI) in patients with bipolar disorder (BD), unaffected relatives (UR) and healthy controls (HC)

	BD^1^	UR^2^	HC^3^	p-value
Total PSQI	8 [5–11]	5 [3–7]	4 [3–6]	< 0.001^1–2^ < 0.001^1–3^ 0.115^2–3^
Duration of sleep (4)	0.0 [0–1]	0.0 [0–1]	0.0 [0–1]	0.803^1–2^ 0.045^1–3^ 0.089^2–3^
Sleep disturbances (5b − 5j)	1.0 [1–2]	1.0 [1–1]	1.0 [1–1]	< 0.001^1–2^ < 0.001^1–3^ 0.332^2–3^
Sleep latency (2 + 5a)	2.0 [1–3]	1.0 [1–2]	1.0 [0–1]	0.007^1–2^ < 0.001^1–3^ 0.022^2–3^
Day dysfunction due to sleepiness (8 + 9)	1.0 [1–2]	1.0 [0–1]	1.0 [1–1]	< 0.001^1–2^ < 0.001^1–3^ 0.364^2–3^
Sleep efficiency	0.0 [0–1]	0.0 [0–1]	0.0 [0–1]	0.012^1–2^ < 0.001^1–3^ 0.126^2–3^
Overall sleep quality (6)	1.0 [1–2]	1.0 [0–1.5]	1.0 [0–1]	0.003^1–2^ < 0.001^1–3^ 0.078^2–3^
Needs meds to sleep (7)	1.0 [0–3]	0.0 [0–0]	0.0 [0–0]	< 0.001^1–2^ < 0.001^1–3^ 0.730^2–3^

PSQI item number is shown in parenthesis. Higher scores equal worse outcome. p-values are denoted as the group between which the comparison is made and calculated based on mean values, using a linear mixed model. Results written as median [interquartile range]. All analyses are adjusted for age and sex

^1^Patients with bipolar disorder (BD)

^2^Unaffected first-degree relatives (UR)

^3^Healthy control persons (HR)

**Fig. 1:**
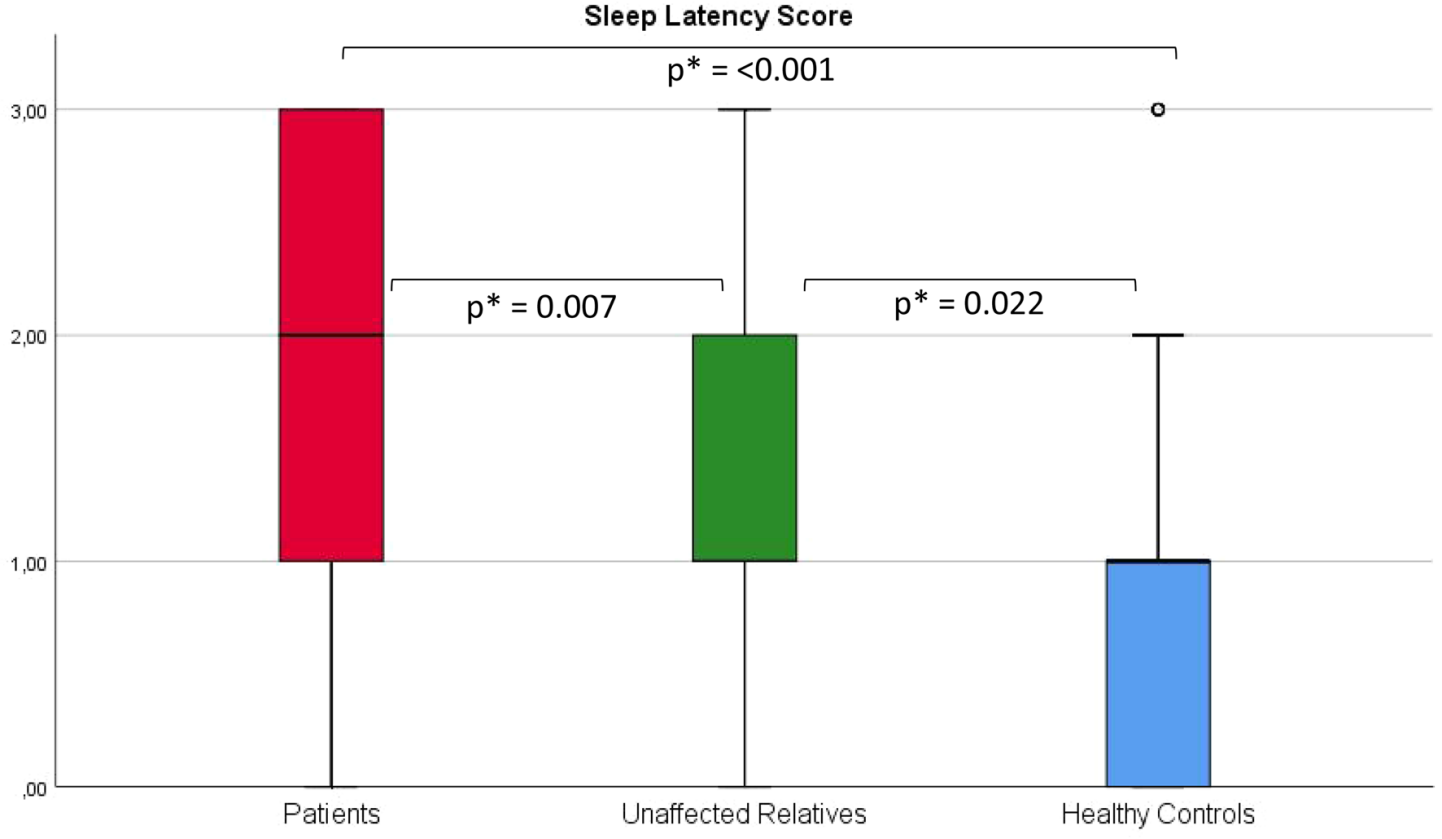
Box plot of the subitem sleep latency from the Pittsburgh Sleep Quality Index in patients with bipolar disorder, their unaffected first-degree relatives and healthy controls. *Statistically significant at the 0.05 level

The UR group trended towards a higher median PSQI score than the HC (5 [3–7] vs. 4 [3–6], p = 0.115). Further analyses, comparing the PSQI subitems, revealed that the UR experienced longer sleep latency than HC (p = 0.022), see Fig. 1. Further, UR reported lower perceived sleep quality (p = 0.078) and shorter sleep duration (p = 0.089) than HC, however only at trend levels, see Table 2.

### Physical activity

**Fig. 2:**
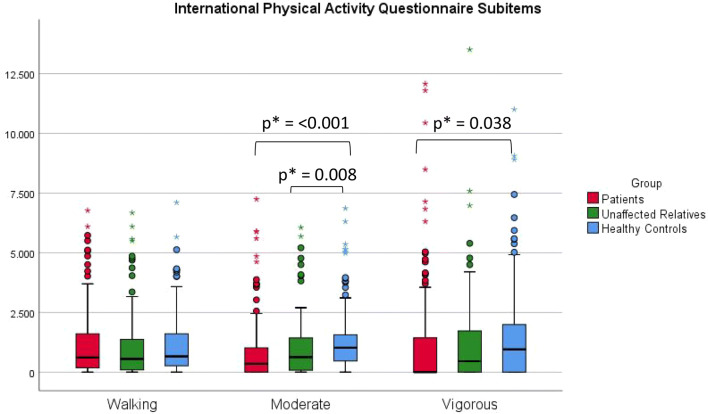
Box plot of three subitems from the International Physical Activity Questionnaire, namely walking, moderate activity and vigorous activity patients with bipolar disorder, their unaffected first-degree relatives and healthy controls. *Statistically significant at the 0.05 level

As can be seen from Table 3, there was a significant difference between the BD and the HC group (2093.8 [980.1–3870.1] MET-minutes vs. 3040.0 [1755.4–5353.7] MET-minutes, p = 0.020), and the UR group showed an intermediary, non-significant pattern compared to HC. The difference was mainly in the vigorous (p = 0.038) and moderate activity (p ≤ 0.001), with walking not differing between BD and HC (p = 0.717, Fig. 2). While the UR group showed an intermediary pattern when looking at median values, they had the highest mean values (median: 2533.7 [936.5–5624.0], mean: 4147.2 CI95: [3367.9–4926.4]). Sedentary behavior was significantly higher in the HC group compared with the BD and UR group. After adjusting for HAMD-17 score, we found no statistically significant differences in IPAQ score between the groups (BD vs. HC: p = 0.304, BD vs. UR: p = 0.155, UR vs. HC: p = 0.603) while the differences remained significant when adjusting for YMRS score (BD vs. HC: p = 0.008, BD vs. UR: p = 0.012, UR vs. HC p = 0.689).

**Table 3:** International Physical Activity Questionnaire (IPAQ) and the physical activity questionnaire from the Copenhagen City Heart Study (CCHS) in patients with bipolar disorder (BD), unaffected relatives (UR) and healthy controls (HC)

	BD^1^	UR^2^	HC^3^	p-value
Vigorous activity (MET-minutes/week)	0 [0–1420]	443.3 [0.0–1781.0]	955.2 [0.0–2000.0]	0.187^1–2^ 0.038^1–3^ 0.747^2–3^
Moderate activity (MET-minutes/week)	336.6 [0.0–1012.0]	632.8 [70.8–1464.0]	1021.2 [449.4–1568.0]	0.008^1–2^ < 0.001^1–3^ 0.531^2–3^
Walking (MET-minutes/week)	603.4 [168.3–1582.4]	556.38 [79.1–1441.44]	656.7 [260.0–1568.0]	0.287^1–2^ 0.717^1–3^ 0.464^2–3^
Sedentary (minutes/day)	300.0 [60.0–480.0]	360 [157.5–480.0]	420.0 [300.0–540.0]	0.749^1–2^ < 0.001^1–3^ 0.020^2–3^
Total IPAQ exercise (MET-minutes/week)	2093.8 [980.1–3870.1]	2533.7 [936.5–5624.0]	3040.0 [1755.4–5353.7]	0.028^1–2^ 0.020^1–3^ 0.713^2–3^
Copenhagen city heart study	3 [2–3]	3 [3–3]	3 [3–3]	0.000^1–2^ 0.000^1–3^ 0.505^2–3^

Results written as median [interquartile range]. All analyses are adjusted for age and sex

*MET* metabolic equivalent of task, a measure for energy expenditure

^1^Patients with bipolar disorder (BD)

^2^Unaffected first-degree relatives (UR)

^3^Healthy control persons (HR)

When removing all participants with HAMD17 and YMRS above 7, the difference between patients with BD and HC on total IPAQ were no longer statistically significant (p = 0.43), neither were the subitems vigorous activity (p = 0.350) and sedentary (p = 0.137). However, the patients with BD still had significantly lower scores on moderate activity (p = 0.044) and on the total Copenhagen City Hearth Study score (p = 0.001).

### Post-hoc analyses

In post hoc analyses, no significant correlations between sleep and exercise when looking at total scores were found. Comparing all subitems of the PSQI and the IPAQ there was a significantly significant statistical correlation between moderate physical activity and total PSQI score (rho = − 0.174, p ≤ 0.001), so more physical activity was correlated with fewer sleep disturbances. Looking separately at patients with BD, a similar however not statistically significant correlation was found between total PSQI and IPAQ (rho = − 0.118, p = 0.096). In the patient group, we further showed a positively significant correlation between HAMD-17 and PSQI (rho = 0.412, p ≤ 0.001) and a negatively significant correlation between HAMD17 and IPAQ (rho = − 0.243, p ≤ 0.001), also when removing the sleep items. Finally, YMRS without sleep items had a significantly positively correlation with total PSQI score (rho = 0.213, p = 0.001), but not with IPAQ.

When investigating the group of patients with BD, we found that age of onset was statistically significantly correlated with total PSQI score (rho = − 0.278, p = 0.0001), pointing towards more sleep disturbances the younger the age of onset. Time with untreated BD was significantly or near-significantly correlated to both PSQI (rho = 0.123, p = 0.081) and IPAQ (rho = 0.150, p = 0.033). The number of previous affective episodes correlated positively with both PSQI (rho = 0.185, p = 0.008) and IPAQ (rho = 0.141, p = 0.043) thus more episodes were correlated with more sleep disturbances and less physical activity.

## Discussion

The present study reported data on sleep disturbances and levels of physical activity from a large sample of patients with newly diagnosed BD in remission or partial remission (n = 227), their UR (n = 76) and HC (n = 148). In accordance with our hypotheses, patients with newly diagnosed BD revealed more sleep disturbances and lower physical activity than the HC. This shows that at the time of diagnosis, patients with bipolar disorder experience sleep disturbances also when they are in partial or full remission. Further, in accordance with our hypothesis, activity levels and sleep disturbances in UR showed an intermediary pattern nonetheless, only the PSQI subitem “sleep latency” differed statistically significantly between UR and HC.

When looking at physical activity we initially found significant differences between BD and UR and between BD and HC, respectively. This was supported by similar differences in the results from the physical exercise questionnaire from the CCHS (Aguib and Al Suwaidi 2015). No differences were identified regarding UR vs. HC. The variance of IPAQ scores in the UR group was larger than among the HC and our lack of findings in this group could be due to the moderate number of participants in the UR group. After adjusting for subsyndromal mood symptoms, the statistically significant differences between patients with BD and HC was reduced to a trend level. This is in line with the statistically significant correlations seen between HAMD-17 and total PSQI and IPAQ scores, pointing towards the impact of having subsyndromal symptoms, thus more depressive symptoms seem to translate into less physical activity and more sleep disturbances. More physical activity seems to impact sleep in a beneficial manner and may be part of the reason why patients with BD exhibit more sleep disturbances.

Surprisingly, the BD group showed the lowest amount of sedentary behavior in contrast to what earlier studies have found (Vancampfort et al. 2016). A partial explanation could be due to actual absence from work or school (at the time of assessment) due to sick leave, in patients with BD. In accordance with prior observations (Kantomaa et al. 2016), we conducted a post hoc correlation analysis and saw a positive correlation between sedentary behavior and educational attainment (rho = 0.187, p ≤ 0.001). Another explanation could be that the BD group underreported their sedentary behaviour, either due to unrealistic ideas about how much they sit or not wanting to reinforce the stigma of living with mental illness (Vancampfort et al. 2017). Thus, the lower reported sedentary behavior in the BD group may be due to inaccurate self-reporting of sedentary activity.

In post hoc correlation analyses we would have expected that more sleep disturbances would be correlated with less physical activity, but this was not the case when looking at total scores in the present sample. Interestingly, we found a negative correlation between moderate physical activity and total PSQI score, suggesting that moderate physical activity could be beneficial for sleep quality. Especially depressive episodes are thought to have beneficial effects from physical activity and this may also be the case for manic episodes, even though the area still lacks well-designed randomized controlled trials (Melo et al. 2016b). In our sample, patients with BD present with a low amount of moderate to vigorous activity, possibly accounting partially for their increased amount of sleep disturbances and their overall higher depression scores.

In a similar study, however not comprising newly diagnosed BD, including 107 patients with BD type I, 74 UR and 80 HC, UR reported longer sleep duration and later time of sleep than HC (Verkooijen et al. 2017). In their case, differences between groups were fully attributed to differences in mood symptoms, and group differences disappeared when adjusting for HAMD-17 and YMRS scores. As stated above, sleep latency remained statistically significantly longer among UR compared with HC in our study, when performing a similar adjustment. Looking at prospective studies of BD offspring four studies have been conducted and over all poor sleepers including those with insomnia predicted onset of BD (Pancheri et al. 2019).

When stating these differences in psychical activity and sleep patterns the impact of medication in the BD group must be considered, as most of the patients received medication at the time of the assessment (87.3%). The medication can unpredictably influence the results in the BD group. Many psychotropic drugs have either sedative or excitatory effects and may affect the amount of physical exercise and sleep. However, the UR group can be observed without the influence of medical treatment.

### Sleep disturbances and physical activity in unaffected relatives

Judging from the hereditability of BD, we would expect around 30–40% of the UR to develop psychiatric illness. If sleep disturbances act as a risk marker for onset of psychiatric illness, we would expect a higher average on the PSQI within the UR group. Our results show that the UR had a trend (p = 0.072) towards reporting more overall sleep disturbances than the HC and reported significantly longer sleep latency, pointing towards that sleep disturbances seems to be a risk or even a trait factor. Two studies including prospective data on high-risk participants (Ritter et al. 2015; Levenson et al. 2015) showed that sleep disturbances seem to be a prognostic indicator of the development of BD in high-risk youth. However, more prospective studies are needed to establish discrete sleep disturbances as risk factor for later onset of BD. Physical activity seems as stated to reduce risk of all-cause mortality thus an increased physical activity in high-risk individuals may act as a protective factor against development of psychiatric illness. Here, the UR group exhibited an intermediary level of physical activity and it is not possible from the present cross-sectional design to pinpoint whether physical activity acts as a protective factor. Nevertheless, the results also point towards that physical exercise have a beneficial impact on sleep, which can be useful as a clinical advice.

### Strengths

First, we recruited a relatively large sample of patients newly diagnosed with BD, their UR and HC. Second, our BD population is recruited from the Copenhagen Affective Disorder Clinic, covering the entire Capital Region of Denmark that offers treatment for patients with newly diagnosed BD. Despite this unique possibility to recruit patients with BD early in their illness course, our findings of illness duration (9 years) and untreated BD (3 years) shows the well-known diagnostic delay in accurately diagnosing BD (Baldessarini et al. 2007). Third, our study profited from high validity of the BD diagnosis, as all patients were initially assessed by a physician specialized in BD and the diagnosis was confirmed by Ph.D. students with a MD or master’s in psychology degree upon inclusion. Fourth, by including first-degree relatives, we provide insight to features possibly preceding BD.

### Limitations

Several limitations must be considered when evaluating our study. First, the HC population was recruited amongst blood donors from the Blood Bank at Rigshospitalet, Copenhagen. This population may be selected for persons healthier than the general population in Denmark (Golding et al. 2013). Second, the sample size of the UR group was modest, so interpretation of our findings in UR should be made with caution. Third, our measure of alcohol the preceding month prior to inclusion may not be representative in patients with BD, as many patients have had a substantial overuse of alcohol preceding initiation of treatment at The Clinic of Affective Disorders, where they at the first visit were recommended alcohol abstinence for 4–12 weeks. As a result, we decided to not adjust for alcohol. Fourth, self-reported physical activity tends to be overreported and the IPAQ has in the last years been under scrutiny for not obtaining the required validity when comparing to objective measures of activity (Lee et al. 2011). However, we would expect this influencing all three groups; hence, the actual level of activity may be lower for all participants. Fifth, the PSQI does not include oversleeping, even though there are ongoing discussions as to whether it can be detrimental (Cappuccio et al. 2010; Gallicchio and Kalesan 2009; Kurina et al. 2013). Sixth, the cross-sectional nature of the design does not allow for studying possible temporal associations between the variables and the self-reported assessment rely on one time point, only. Finally, the present results are based on self-reported sleep questionnaires and not objectively measured sleep patterns, e.g. using telemonitoring devices which is recommended in future studies.

### Conclusion

Patients with BD in full or partial remission reported more sleep disturbances and less moderate and vigorous physical activity than their UR and HC. The sleep disturbances and lower activity levels were associated with subsyndromal affective symptoms pointing towards the clinical importance of obtaining full remission in BD. The unaffected first-degree relatives reported sleep latency problems which may possibly be a risk marker of BD and they also used more time on moderate to vigorous physical activity. In the ongoing longitudinal BIO study, we will obtain further knowledge about sleep disturbances and patterns of physical activity preceding BD.

## Data Availability

The datasets used and/or analysed during the current study are available from the corresponding author on reasonable request.
